# Biopolymers as a sustainable solution for the enhancement of soil mechanical properties

**DOI:** 10.1038/s41598-019-57135-x

**Published:** 2020-01-14

**Authors:** Antonio Soldo, Marta Miletić, Maria L. Auad

**Affiliations:** 1 Graduate Student, Department of Civil Engineering, Auburn University, Auburn, AL 36849-5337 USA; 2 Department of Civil, Construction, and Environmental Engineering, San Diego State University, San Diego, CA 92182-1324 USA; 3 Department of Chemical Engineering, Auburn University, Auburn, AL 36849-5337 USA

**Keywords:** Environmental sciences, Civil engineering

## Abstract

Improving soil engineering properties is an inevitable process before construction on soft soil. Increasing soil strength with chemical stabilizing agents, such as cement, raises environmental concerns. Therefore, sustainable solutions are in high demand. One of the promising solutions is the usage of biopolymers. Five biopolymer types were investigated in this study: Xanthan Gum, Beta 1,3/1,6 Glucan, Guar Gum, Chitosan, and Alginate. Their effect on the soil strength improvement was experimentally investigated by performing unconfined compression, splitting tensile, triaxial, and direct shear tests. All tests were performed with different biopolymer concentrations and curing periods. Additionally, in order to have an insight on the susceptibility to natural elements, plain soil, and biopolymer-treated specimens were exposed to real atmospheric conditions. The extensive experimental results showed that the soil strength tends to increase with the increase of biopolymer concentration and with the curing time. However, it was shown that the soil strength does not considerably change after a certain biopolymer concentration level and curing time. Furthermore, it has been observed that the biopolymer-treated specimens showed better resistance to the influence of the environmental conditions. In general, Xanthan Gum, Guar Gum, and Beta 1,3/1,6 Glucan showed the most dominant effect and potential for the future of sustainable engineering.

## Introduction

Rapid population growth and urbanization often cause the need to build over soft and unfavorable soil present in adverse surroundings. This further urges the need to improve the originally non-favorable soil. Soil improvement technologies can be divided into three major groups: mechanical, biological and chemical soil improvement technologies. The most common chemical stabilizing agents that are used for chemical soil stabilization are cement and lime. However, their use raises many environmental concerns such as CO_2_ emissions due to cement production^1^, prevention of vegetation growth, groundwater contamination, and heat island creation, to name a few. In fact, in 2002, the production of cement contributed about six percent to the world’s CO_2_ emission^2^, and Andrew^1^ had pointed out the possible increase of that number in more recent years. Therefore, the demand for sustainable and environmentally friendly solutions for ground improvement is in high increase.

Biological approaches are emerging in the field of geotechnical engineering and techniques like microbial induced carbonate precipitation (MICP) have shown to be an effective means of effectively improving soil strength and the load-bearing capacity^3–7^. However, these biological approaches require the introduction of a large microbial community and cementation reagents to the soil to create a highly specialized growth environment for the bacteria which may result in the generation of effluent ammonia. Furthermore, the MICP method is limited to the coarse-grained soils due to microbe infiltration problems. This is because the pores of the fine-grained soils are too small to provide an appropriate bacteria growth environment^8^. Therefore, it is desirable to seek a non-microbe, but still bio-inspired and sustainable ground improvement solution.

An attractive alternative to MICP is the soil improvement with biopolymers because it does not require microorganism’s cultivation in the soil^8^. Biopolymers are organic polymers that are produced by different biological organisms^7^. In nature, biopolymers can be found in large amounts. They are biodegradable and have no negative effects on the environment; therefore, they might be favorable soil-improvement material^7^. Also, unlike MICP, biopolymer treatment can be used for the improvement of fine-grained soil^9,10^. An additional reason why biopolymers have an advantage over MICP is the fact that they do not require any nutrient injection and can be directly used for ground improvement.

According to the previous research, biopolymers, such as Guar Gum (GG), Xanthan Gum (XG), Chitosan (CHI), and Beta 1,3/1,6 Glucan (BG), can significantly improve engineering properties of soils^11–16^. The effect of XG on soil properties was investigated by various researchers and it was found that XG can considerably increase the compressive and shear strength of soils, especially of soils that contain a significant amount of fine-grained aggregates^14,17,18^. In addition, XG proved to decrease the settlement of collapsible soils^19^. GG, aside from soil strength improvement, can also be used for sand stabilization, liquid shoring^20,21^, and the reduction of soil compressibility^19^. Chang & Cho^22,23^ showed that BG can increase the compressibility of soil, as well as the plasticity index and the compressive strength. CHI can improve soil strength, but its effect decreases with the reduction of water^16^. Therefore, the additional strength that could be achieved with the addition of CHI to the soil fades as the soil becomes dry. Alginate (ALG) is a biopolymer that was extensively used in biomedical industries^24^, but its potential for soil improvement has not been well investigated yet.

Nevertheless, even though biopolymers show substantial environmental and technological contributions to the improvement of soil engineering properties, biopolymers are still underutilized in geotechnical engineering practice. Some of the main reasons are the lack of adequate characterization of their engineering behavior and the lack of analysis methods that engineers could use to incorporate biopolymers into their designs. Therefore, the main objectives of this research are to develop an eco-friendly, biopolymer-based soil improvement and to investigate the effect of biopolymers on soil strength. In the presented study, five types of biopolymers (XG, BG, GG, CHI, and ALG) with different biopolymer concentrations were investigated (1%, 2%, and 4%). Furthermore, since curing time and water content are some of the key factors that influence the strength of soil, the relation between them and the strength of the biopolymer-improved soil was observed.

## Materials and Methodologies

### Base soil

**Figure 1:**
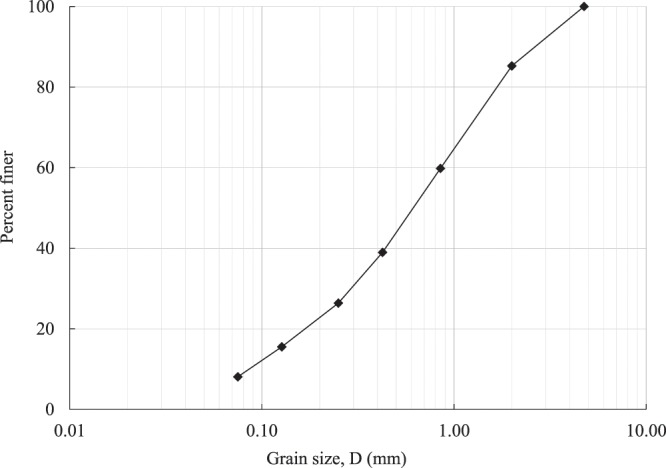
Grain size distribution of the base soil.

Residual Piedmont soil was selected as the base soil for this study because of its significant presence in the east and south-east part of the United States. Figure 1 depicts the grain size distribution curve for Piedmont residual soil used for this study. Grain size distribution curve yielded the results for the coefficient of curvature and coefficient of uniformity as 1.5 and 10, respectively. The soil had eight perfect fine particles with the liquid limit, plastic limit and the index of plasticity being 49, 29, and 20, respectively. Therefore, according to the Unified Soil Classification System, fine particles are classified as silt with low plasticity, and the overall classification of residual Piedmont soil that was used in this study was SW-SM (well-graded sand with silt).

### Biopolymers

Five different biopolymers were used in this study and their properties are described in the text below.

#### Xanthan gum

XG is a polysaccharide made by the fermentation of a carbohydrate-source medium e.g. glucose. Xanthomonas campestris bacterium is the bacteria which induces the fermentation process that creates XG^25^. XG can be dissolved in hot and cold water and it also increases the viscosity of the medium it is mixed in. Solutions containing XG are non-Newtonian with high pseudoplasticity, which means that their viscosity decreases with an increase in shear stress^25^. Nowadays, it can be found in the cosmetic industry, food industry, agriculture^25^, oil drilling industry^26^, and civil engineering^18^.

#### Guar gum

GG is a polysaccharide extracted from guar beans or guar, officially known as Cyamopsis Tetragonolba. Like XG, it can dissolve in hot and cold water. Because solutions with GG act as gels or binders, it is found in various industries e.g. cosmetic industry, food industry^27^, oil and gas drilling industry^28^, and in civil engineering^18^.

#### Beta 1,3/1,6 glucan

BG is a carbohydrate made of glucose molecules that are bound by glucopyranose^29^. Yeast, fungi, cellulose, cereals and some bacteria contain BG^30^. Like XG and GG, BG dissolves in water at any temperature and creates gelatinous solutions^30^. This type of biopolymer is being extensively investigated for its potential for providing health improvement benefits^29,31^. Furthermore, it has been used in civil engineering^22,23^.

#### Chitosan

Amino polysaccharide chitosan can be obtained by deacetylation of chitin. Chitin is a biopolymer abundant in nature and it is contained in shells. CHI is characterized by hydrophilicity, biocompatibility, biodegradability, and non-toxicity^32^. Currently, CHI is present in the cosmetic industry, pharmaceutical industry, agriculture^33^, food industry, and in civil engineering^16^.

#### Alginate

ALG is an anionic biopolymer and is usually obtained from brown seaweed e.g. Laminaria japonica, Macrocystis pyrifera, Ascophyllum nodosum, Laminaria hyperborean, etc^34^. It is characterized by low toxicity, relatively low cost, biocompatibility, and mild gelation when mixed with divalent cations. Therefore, it is being investigated for more different applications while mostly being used for biomedical purposes^24,35^.

### Specimen preparation

All mixing quantities were batched using an electronic balance and mixed with the stainless-steel stirrer. In the mixing process, two approaches were taken. In the first approach, all dry components (base soil, and biopolymer) were first slowly mixed together for a few minutes. Four types of biopolymers (XG, GG, CHI, and BG) were mixed into the soil with three different concentrations (1%, 2%, and 4%) with the respect to the mass of the base soil. After mixing a biopolymer with soil, water was sprayed in the soil-biopolymer mixture up to 16.5% of the mass of the base soil. Water was the most important factor in the preparation of the specimens. The water content of 16.5% corresponded to the optimum water content of the soil and it was selected as relatively best for the specimen preparation. Lower water contents produced non-uniform soil samples, whereas higher water contents increased the softness of specimens which hindered the specimen preparation. While adding water, the soil-biopolymer mixture was continuously mixed to ensure homogeneous water distribution.

ALG was the only biopolymer that was treated in a different manner. The reason for that was the fact that ALG required a binding agent, such as calcium chloride, which has the ability to hold the ALG chain-molecules together. Therefore, alginate was first dissolved in water (13.5% of the mass of the base soil), as well as calcium chloride (3% of the water of the mass of the base soil). In this case, water was not sprayed into the soil, but the dissolved alginate was mixed with the base soil. After that, the dissolved calcium chloride was added into the mixture. In addition, it was noticed that the dissolving process was not the same for all ALG concentrations. The lower concentrations of ALG in the ALG-water solutions were more uniform than higher concentrations. Therefore, the higher ALG concentration (4%) was omitted from the research due to the bad workability of the 4% ALG-water solution.

**Figure 2:**
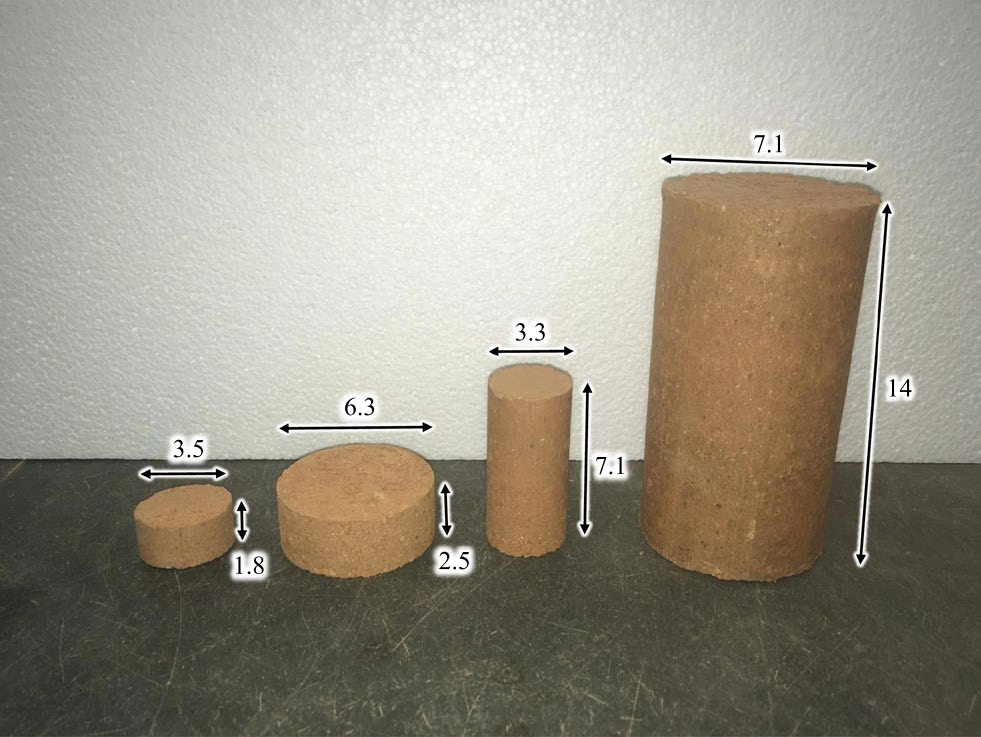
Photo of the splitting tensile test (left), direct shear test (middle-left), the unconfined compressive test (middle-right), and the triaxial test (right) specimens with their dimensions (all dimensions are in centimeters).

Finally, once the dry components were fully mixed with the liquid part, the blends of soil, biopolymer, and water were placed in cylindrical molds and evenly compacted. The mold for the specimens which were tested for unconfined compression had a diameter of 3.3 cm and a height of 7.1 cm. Specimens were compacted in five layers with a standardized hammer. Each layer was compacted by applying pressure twenty-five times on the surface of the soil that was in the cylinder. The mold that was used to make the specimens for the splitting tensile strength test had a diameter and height of 3.5 and 1.8 cm, respectively. The mold was filled with soil and compacted in three layers with twenty-five hits for each layer. Specimens for the triaxial test were compacted in five layers in a mold with a diameter of 7 cm and a height of 14 cm. The mold used for the direct shear test had a diameter of 6.3 cm and a height of 2.5 cm. The soil was compacted in three layers where each layer was compressed approximately twenty-five times. After compaction, specimens were extruded from the molds (Fig. 2) and left to cure for a specific amount of time. Specimens for the unconfined compression and splitting tensile test were tested after one hour, five days, and 30 days of the preparation. Additionally, specimens with 1% XG, GG, and BG were tested under uniaxial compression conditions every 24 hours during the first five days after the preparation. Triaxial tests and direct shear tests were performed after five and 30 days of curing.

Molds for the unconfined compression and splitting tensile strength tests were also used to make specimens that were investigated under the influence of the environment. After preparation, those specimens were first left to dry in the laboratory conditions for 10 days after which they were placed in the open field to go through cycles of wetting and drying for 30 days. For this type of experiment, specimens were made of plain soil and soil with 2% XG.

### Mechanical testing

Unconfined compression (UC), splitting tensile (ST), unconsolidated undrained triaxial (UU), and direct shear (DS) tests were carried out to determine and analyze the mechanical properties of specimens made of plain and biopolymer-treated soil. After compaction and extraction from the molds, one portion of specimens was tested within an hour of preparation (wet specimens) and other specimens were tested after designated curing time.

#### Unconfined compression test

The uniaxial or unconfined compression test also known as a cylinder compression test is the appropriate test for determination of the compressive strength of a cohesive soil regardless of the amount of biopolymers that it may contain. For determination of compressive strength, the axial load was applied with a strain rate of 1.5%/min, as per American Society for Testing and Materials Standard (ASTM) ASTM D2166/D2166M^36^. The compressive strength of the specimen was computed by dividing the maximum load attained during the test by the cross-sectional area of the specimen. Furthermore, in order to obtain Young’s modulus, *E*, of a sample from an unconfined uniaxial compressive test, the applied load, and a longitudinal deformation were recorded. Young’s modulus was extracted from the slope of the straight line passing through the origin.

#### Splitting tensile strength test

A direct tension test is the most direct way of collecting the information needed for the determination of material tensile properties experimentally. However, direct testing of the tensile strength of soil comes with a lot of issues regarding boundary conditions and the fragility of the material. Therefore, a quicker and simpler alternative to the direct tension test is an indirect ST test. Its advantages over the direct tension test are that it is fairly easy to conduct, it has already been standardized, which makes it a cheap and highly applicable test in practice. ST tests were performed following general procedures described in the ASTM D3967-16^37^ but with a modified apparatus to accommodate small specimen sizes and relatively low loads. The load was applied at the constant strain rate of 1.5%/min. It must be noted that an ST test does not give direct soil tensile properties under tensile loading. Thus, the tensile strength was back-calculated from the maximum compressive load and the dimension of the specimens using the following formula:

1 $${\sigma }_{T}=\frac{2P}{\pi LD}$$

where *σ*_*T*_ is tensile strength; *P* is a compressive force at failure; *D* is the diameter of the specimen; *L* is specimen thickness.

#### Undrained unconsolidated triaxial test

One of the advantages of triaxial tests is that they can be performed under a broad range of confining pressures. That means that triaxial tests can be used, to some extent, to replicate the stress conditions in soil that are present in the field. UU test is the fastest triaxial test which can be used to find the maximum deviatoric stress and undrained shear strength at different confining pressures. In this study, UU was performed in accordance with ASTM D2850-15^38^. The specimens were placed in a plexiglass chamber under the confining pressure of 100 kPa. During the shearing stage of the test, the axial strain was applied under the rate of 0.7%/min. UU tests were carried out after five and 30 days of curing. From the preliminary data collected by the authors, it was evident that XG, GG, and BG showed the most promising results for the improvement of soil strength. Therefore, only those three types of biopolymers were investigated under the UU test.

#### Direct shear test

The DS test is commonly used to determine the cohesion and internal friction angle of soil. Those shear strength parameters cannot be obtained from UC, ST, and UU tests. Consolidated drained triaxial test, and consolidated undrained triaxial test can provide cohesion and friction angle, but they are not suitable for biopolymer-treated soil specimens. The DS test was performed in accordance with ASTM D3080-11^39^. The shearing was conducted under normal loads in the range between 100 kPa and 760 kPa. The rate of shearing displacement was set as 0.06 cm/min. Since it was concluded from the preliminary UC and ST test results that XG, GG, and BG had the most dominant effect on the soil strength, the DS tests were conducted only on the soil specimens improved with these three biopolymer types. The tests were conducted after five and 30 days of curing.

### Scanning electron microscope imaging

Soil-biopolymer interaction varies depending on the engaged types of soil and biopolymer. For instance, fine-grained particles and biopolymers have electrically charged surfaces which creates an electrostatic bond between them^18^. The electrical surface charge in coarse-grained soils is practically non-existent; thus, the bond that is created between coarse-grained material and biopolymer can be presented as a thin film wrapped around coarse particles^9^. In this study, a Scanning Electron Microscope (SEM) Zeiss EVO 50 was used to closely observe the interaction between the silty sand and different types of biopolymers. Small fractions of biopolymer-treated soil, with a size of 0.2–0.3 cm^3^, were glued to a carbon tape on the top of aluminum stubs. The specimens were then coated with a thin layer of gold to prevent charging of the analyzed surface, to promote the emission of secondary electrons so that the specimen conducts evenly, and to provide a homogeneous specimen surface for analysis and imaging.

## Results and Discussion

Several samples that were prepared in the same manner were tested in each test, and the average values of the results are presented in this chapter.

### Unconfined compression test

**Figure 3:**
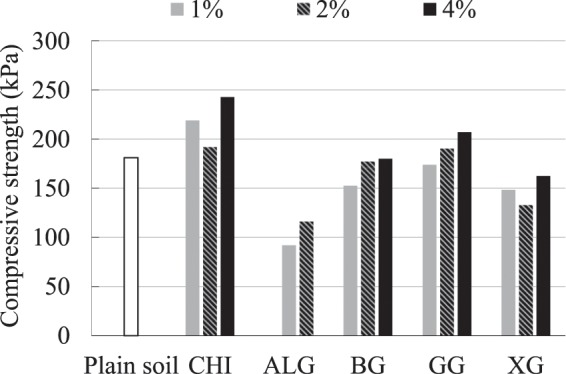
Influence of different type biopolymers and biopolymer concentration on the compressive strength of the specimens tested after one hour of curing.

Soil with different admixtures showed different strengths when tested in the UC test. Figure 3 shows the influence of different types and different concentrations of biopolymers on the compressive strength of the wet specimens which were tested within an hour of the preparation. It can be seen that for the biopolymer-treated specimens, an increase in the biopolymer concentration resulted in the increase of its compressive strength. The only series of specimens that did not follow this trend was with 2% XG and 2% CHI. ALG-treated specimens stand out with the lowest achieved compressive strengths as a result of their specific way of preparation. Even though Fig. 3 shows a variety of achieved compressive strengths, further research showed that the change of strength that happens within one hour is not as substantial as the change that happens over a longer period of time. This indicates that within one hour of the preparation, the strength of wet specimens depended more on the base soil strength and less on the soil-biopolymer bond. This is because the biopolymers within one hour were still in a gelatinous form with low strength. Similar behavior was reported by Swain *et al*.^40^ where a dispersive soil was treated with various concentrations of XG and GG.

**Figure 4:**
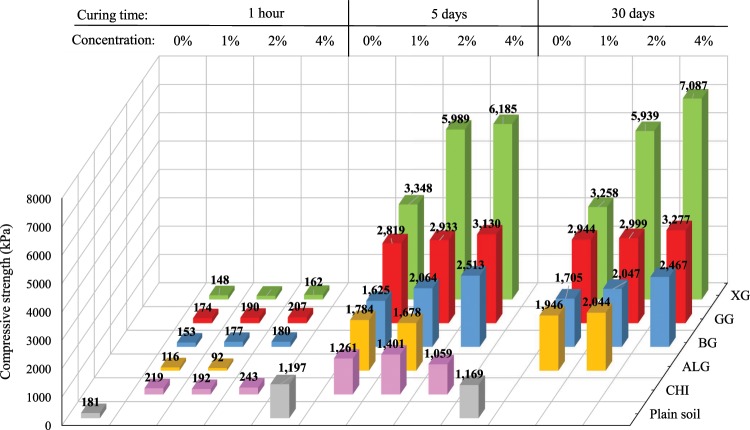
Change in compressive strength under the influence of time, biopolymer-type, and biopolymer concentrations.

Figure 4 depicts the impact of different biopolymer types, biopolymer concentrations, and curing time periods (one hour, five days, and 30 days) on the compressive strength. It is evident from the plot that the soil compressive strength considerably improved with time and biopolymer concentration. These findings coincide with other research studies that showed as well that the compressive strength of soil increases with the increase of biopolymer concentration and curing time of biopolymer-treated soil^15,18,41^. Biopolymers in soil harden with drying which further improves soil strength. XG had the highest impact on the increase of compressive strength. After five days of curing, CHI did not lead to any further increase in the compressive strength even though the specimens with CHI had the highest compressive strength right after the mixing. Therefore, CHI was excluded from further research of this type. The reason for the CHI’s low effect on compressive strength was the fact that it requires acidic water to reach the maximum effect. The usage of the acidic water was avoided in this study because of its possible negative effect on the environment. Furthermore, ALG solution showed a promising effect on strength improvement. However, the difference in the compressive strengths between ALG samples with different concentration levels was not high. Even though the higher concentration of ALG might have a larger impact on the improvement of the compressive strength, the workability at the higher concentrations of ALG can restrict the amount of ALG which can be applied to the soil. Specimens modified with BG, GG, and XG biopolymers showed the most dominant effect on the compressive strength with the XG being the most promising.

In addition, after comparing the achieved compressive strengths after five and 30 days of curing for all biopolymer-mixtures, it can be concluded that the strength did not go under major changes in the period between five and 30 days. Thus, it can be stated that the majority of the compressive strength developed within the first five days after the preparation. Similar behavior was reported by Cabalar *et al*.^42^ where XG-treated soil after three days achieved more than 70% of the compressive strength that was recorded after 28 days.

**Figure 5:**
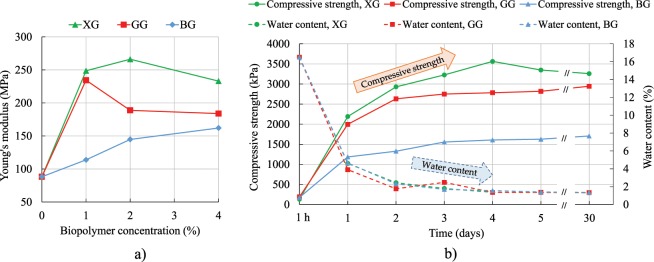
(**a**) Influence of biopolymer type and concentration on Young’s modulus (after five days of curing), (**b**) Change in the average compressive strength and water content during 30 days of curing.

The presence of biopolymers increases Young’s modulus (modulus of elasticity, *E*) of soil in a nonlinear manner. Figure 5a depicts the change of *E* with the different concentrations of XG, GG, and BG after five days of curing. It can be seen that there is an optimum concentration at which *E* reaches its peak value. The optimum biopolymer concentration changes depending on the biopolymer type. From the experimental data, the optimum biopolymer concentration for GG, and XG is approximately around 1%, and 2%, respectively. Unlike for XG and GG, the optimum biopolymer concentration of BG cannot be well predicted from the obtained results and it might be past 4%. These results support the hypothesis of the existing optimum concertation of biopolymers which varies with the type of biopolymers and should be considered when utilizing biopolymers for soil stabilization. Also, Ayaldeen *et al*.^19^ investigated the effect of GG and XG on Young’s modulus of silt. The concentration levels of biopolymers ranged between 0.25% and 2%. The results indicated that the optimum concentration for GG was between 1% and 2%, while in the case of XG, their results suggested that the optimum concentration was not under 2%.

Additional testing was performed in order to closely determine the time needed for the specimens to achieve their compressive strength. The specimens with 1% XG, GG and BG were mixed in the previously described manner and tested for UC (Fig. 5b). Tests were performed an hour after the preparation and every 24 hours during a period of five days. As stated previously, moisture content has a major effect on soil strength. Thus, after each test, specimens were placed in the oven (110 °C) for the purpose of measuring the moisture content. Figure 5b shows that the decrease in water content corresponds to the increase in the strength of the soil. Furthermore, it can be noted that most of the strength developed after three to four days of curing. After that time period, water content and compressive strength became constant. The inverse proportionality of water content and compressive strength is a typical relationship that was reported in other research as well^18,22^.

### Splitting tensile strength test

**Figure 6:**
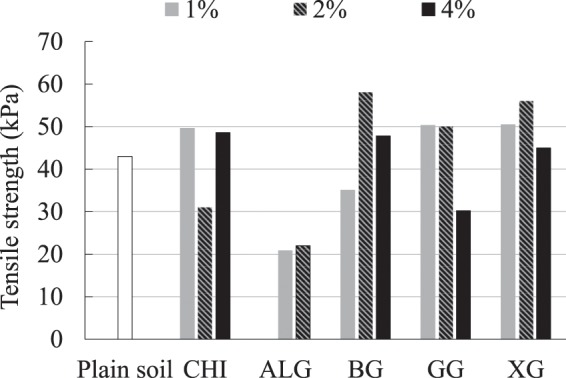
Influence of biopolymers on the tensile strength of the fresh specimens.

The increase of soil tensile strength with the addition of XG and GG was proven by direct tensile tests performed by Muguda *et al*.^43^. They have found that the tensile strength of soil increases with biopolymer concentration and time. Furthermore, Ma, H. & Ma, Q.^44^ also showed an increase in the tensile strength of soil with the increase of sodium carboxymethyl cellulose concentration. In this study, during the first hour after the preparation, all tensile strengths were in the range of 20–60 kPa, and biopolymer-improved soil tensile strengths did not show a major deviation from the tensile strength of the plain soil (Fig. 6). Specimens made with ALG stood out with the lowest tensile strength. This is most likely due to the different preparation method of ALG-improved specimens, which accentuates the importance of the preparation method. Similar to the UC test results, one hour curing time was not long enough to develop a strong soil-biopolymer bond.

**Figure 7:**
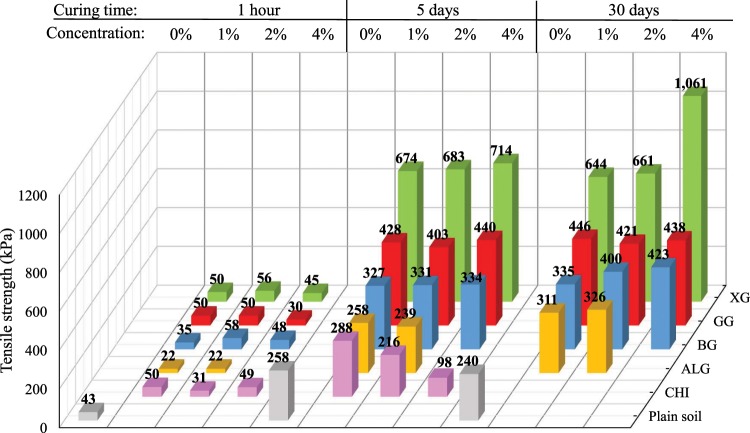
Change in compressive strength under the influence of time, biopolymer-type, and biopolymer concentration.

Figure 7 shows the effect of different types of biopolymers, biopolymer concentration, and curing time on the soil tensile strength. It is evident that the strength is considerably higher after five and 30 days than the strength which was achieved within one hour after preparation. Thus, once again, it can be concluded that biopolymers need curing time to achieve their potential. CHI showed the most negative influence on the tensile strength of soil, which happened mostly due to the loss of water. The concentration of 4% CHI had an especially negative effect on the tensile strength. Therefore, CHI was excluded from further research on tensile strength. ALG, BG, GG, and XG showed a positive effect on the tensile strength after five and 30 days but XG had the most dominant effect. As in the case of the unconfined compression, most of the strength was achieved during the first five days.

### Unconsolidated undrained triaxial test

**Figure 8:**
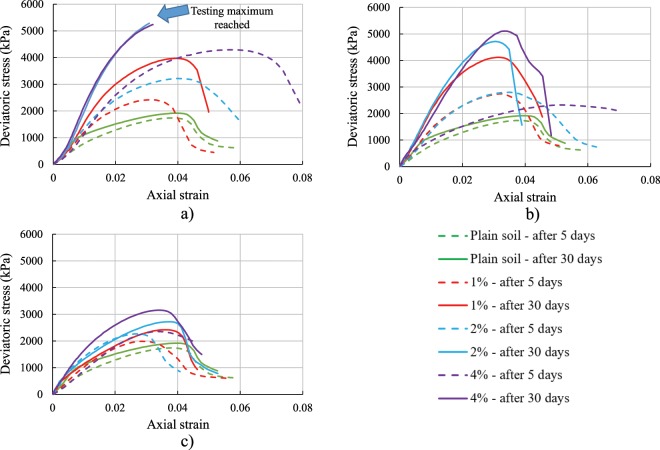
Experimental deviatoric stress versus axial strain. UU testing plots for different curing periods, biopolymer concentrations, and type of biopolymer: (**a**) XG, (**b**) GG, (**c**) BG.

Figure 8 shows the experimental stress-strain responses obtained from the UU triaxial tests on plain and biopolymer-reinforced specimens. Only those polymers that have achieved the most promising results in the prior testing were used in the UU testing program, namely, BG, GG, and XG. All specimens were tested five and 30 days after preparation. Unlike UC and ST test results, the difference in the maximum deviatoric stress of specimens tested after five and 30 days of preparation was notable. The reason for that is the fact that larger specimens need more time for water content to decrease while drying, and the soil strength increases with the loss of water content. Specimens for UC test and ST test had a volume of 60.7 cm^3^ and 17.31 cm^3^ respectively, but the specimens for UU test had a volume of 538.51 cm^3^. Furthermore, this difference in the maximum deviatoric stress was more noteworthy for biopolymer-reinforced soil than it was for the plain soil. It was found that the improvement in strength during the period between five and 30 days for the plain specimens was 10%.

For the soil specimens amended with XG, the concentration of 1% caused the increase in the maximum deviatoric stress by 66% between five and 30 days, whereas concentrations of 2 and 4% caused an increase of 64 and 22% for the same period, respectively (Fig. 8a). It is important to notice that after 30 days the maximum deviatoric stress of the specimens made with 2% and 4% XG was higher than the testing machine capacity. Therefore, the highest measured values were taken as the maximum achieved values.

The addition of 1% GG resulted in the improvement of the maximum deviatoric stress by 51%, and for the specimens with 2% GG the improvement was 68% (Fig. 8b). In addition, after five days of curing, specimens with 4% GG had lower peak deviatoric stress than the specimens with 1 and 2% GG. This might be related to optimum biopolymer concentration. A similar effect of the optimum biopolymer concentration was presented in Fig. 5a where Young’s modulus of soil started decreasing for the concentrations higher than 1% GG. After 30 days, the maximum deviatoric stress of soil reinforced with 4% GG increased by 120% in comparison to the maximum stress that was achieved after five days.

Similar as in the cases with XG and GG, BG also increased the maximum deviatoric stress of the specimens that were tested in the UU test. In the period between five and 30 days, specimens with 1, 2 and 4% BG had improved for 22, 20 and 34%, respectively (Fig. 8c).

These findings are consistent with previous research studies of biopolymer-treated soil under triaxial loading conditions showing that biopolymers can increase the peak deviatoric stress^40,42–46^. On the other hand, Karimi^47^ showed that the maximum deviatoric stress of 1% XG-treated silt can decrease in the period between five and 30 days. Even though consolidated triaxial tests were frequently used to demonstrate the strength of soil, the authors recommend using unconsolidated undrained tests for testing of biopolymer treated soil. The reason behind that is that biopolymers tend to dissolve in water^48^. Therefore, adding water to biopolymer treated soil would hinder the strength of the soil.

### Direct shear test

Previous research showed that the change in cohesion and the friction angle is depended on biopolymer and soil types. Cho and Chang^49^ used the direct shear test to investigate the effect of different concentrations of gellan gum on the cohesion and friction of sandy and clayey soil. Their results indicated that the cohesion increased with the increase of gellan gum concentration. The friction angle increased with the increase of gellan gum for the pure clay and for the sand that had between 20% and 50% of clay. However, the change in the friction angle of pure sand was found negligible. Khatami & O’Kelly^40^ showed that agar and starch increase the cohesion of sand but reduce the friction angle. Ayeldeen *et al*.^19^ demonstrated with the direct shear test that XG and GG increase the cohesion of a soil that had a significant presence of fine particles, but the biopolymers slightly decreased the friction angle that soil.

**Figure 9:**
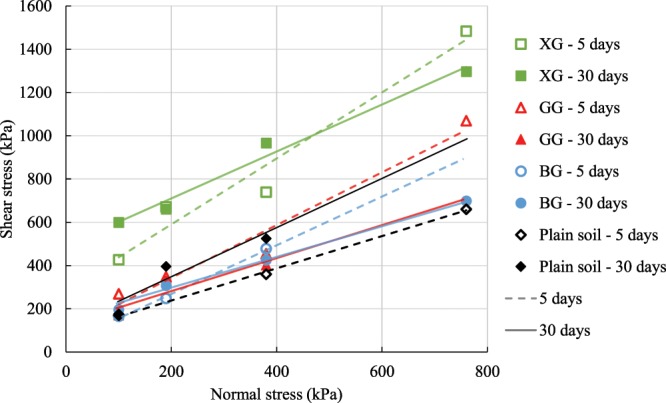
Strength envelopes from DS test results.

In the present study, the main objective was to quantify the change in cohesion and the friction angle due to the influence of XG, GG, and BG for two different curing times. The strength envelopes from DS test results are depicted in Fig. 9. The slopes of the plotted lines relate to the friction angles, and the intercepts with the vertical, shear stress axis represent the cohesion. The DS results are summarized in Table 1.

**Table 1:** Influence of biopolymers and time on the cohesion and friction angle of soil.

Curing time	Plain soil		XG		GG		BG	
	Cohesion (kPa)	Friction angle (°)	Cohesion (kPa)	Friction angle (°)	Cohesion (kPa)	Friction angle (°)	Cohesion (kPa)	Friction angle (°)
5 days	89	36	285	57	98	51	44	48
30 days	107	49	493	47	131	37	144	36

Specimens exposed to atmospheric conditions.

The plain soil had a cohesion of 89 kPa and the angle of friction of 36° after five days of curing. The cohesion increased by about 20 kPa in the period between five and 30 days. The soil reached a high friction angle through a period of 30 days because of its highly compacted state.The addition of the XG tripled the cohesion of the plain soil five days after the preparation. After 30 days, XG-treated soil had the cohesion of almost 500 kPa. The friction angle of XG-treated soil was 57° after five days of curing, but it dropped by 10° in the period between five and 30 days and it was close to the friction angle of the plain soil.

After five and 30 days of curing, the cohesion of GG-treated soil was 9 and 24 kPa higher than the cohesion of the plain soil, respectively. However, for the period between five and 30 days of curing, the friction angle dropped from 51° to 37° which was lower than the friction angle of the plain soil.

The cohesion of BG-treated soil, after five days, was half of the cohesion of the plain soil, but the cohesion increased by 100 kPa between five and 30 days after curing. In the same period, the friction angle reduced from 48° to 36° which was lower in value than the final friction angle of the plain soil.

This research concludes that XG, GG, and BG have the tendency to increase the cohesion of silty sand, but ultimately reduce the friction angle of the silty sand. The increase of cohesion is the result of the binding properties of biopolymers that glue the soil particles together. However, biopolymer formations tend to smoothen the surface of coarse particles which results in reducing the friction between soil particles and the overall friction angle of the soil^50^.

### Specimens exposed to atmospheric conditions

**Figure 10:**
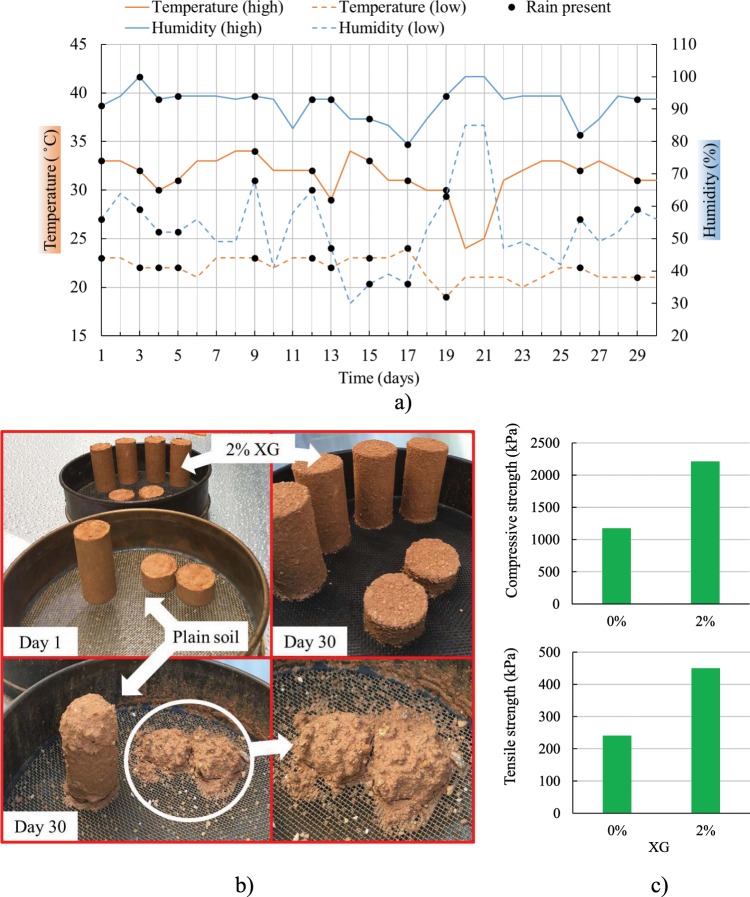
(**a**) Daily temperature and relative humidity measured in the field, (**b**) Specimens exposed to atmospheric conditions, (**c**) Strength of the specimens with 2% XG (after being exposed to atmospheric conditions) compared with the strength of plain soil specimens which were kept in the laboratory for 30 days.

Nine samples were fully exposed to the influence of the environment. Daily temperature and relative humidity measured in the field are summarized in Fig. 10a. It can be noted from Fig. 10b that the XG-improved specimens showed higher wetting-drying durability under the atmospheric influence. Plain specimens were severely damaged after 30 days and could not be tested for UC nor ST tests. Specimens with 2% XG kept their shape for the entire 30 days. Thus, specimens with 2% XG were tested for UC and ST tests. Figure 10c shows the comparison in the strength of the biopolymer-reinforced specimens (with 2% XG) exposed to atmospheric conditions and the strength of plain specimens tested after 30 days of curing in the laboratory. It is evident that the treated specimens had higher strength even after being exposed to the influence of the environment.

### SEM analysis

**Figure 11:**
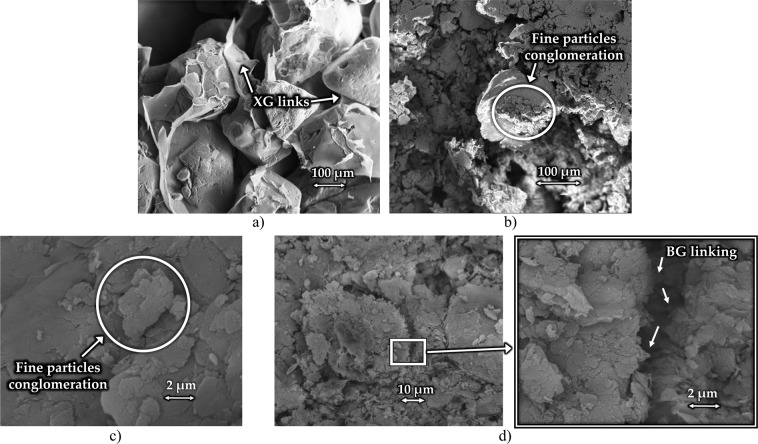
(**a**) XG with coarse-grained soil47; (**b**) GG with silty sand; (**c**) XG with silty sand; (**d**) BG with silty sand.

For comparison purposes, Fig. 11a shows the interaction of an XG biopolymer and pure sand from the author’s previous studies^46^. The formation of XG can clearly be seen as a coating agent surrounding coarse-grained particles and bridging the particles that are not in direct contact. Figure 11b depicts the SEM image of the GG-treated silty sand from this study. Due to the presence of the fine-grained particles in the soil, the clear biopolymer coating formation cannot be seen. GG and fine particles formed a dense structure that is attached to the larger sand particles. This tendency of biopolymers to create conglomerates together with the fine particles is presented in Fig. 11c as well, where lumps of fine particles were formed due to the presence of XG. To observe biopolymer-fine grained particle interaction, the magnification had to be more than 30 times higher than for the pure sand with XG. The high presence of fine-grained soil makes the biopolymer linkages more difficult to see on SEM images. Figure 11d shows a thin formation of BG that is visible due to the detachment of two soil masses of the silty sand. The thin foil that is spreading in the crack in Fig. 11d is BG linking.

Observation of the biopolymer-soil interaction leads to two main conclusions. Biopolymers act as a coating agent on coarse-grained soil and can be easily observed under SEM. On the other hand, in sand-silt mixtures biopolymers create, through the electrostatic bond, conglomerates of fine particles that attach to the larger coarse-grained particles. A formation like that requires more detailed observation under SEM.

## Conclusion

The main objectives of this research were to develop an eco-friendly, biopolymer-based soil improvement technique and to experimentally investigate the biopolymer effect on the soil mechanical properties. In order to achieve these objectives, five different biopolymer types (XG, BG, GG, CHI, and ALG), three different biopolymer concentrations (1%, 2%, and 4%), and several different curing times were investigated. Four mechanical strength tests were performed: UC, ST, DS, and UU tests.

Since curing time and water content are some of the key factors that influence the biopolymer-soil strength, the relation between them and the strength of the biopolymer-improved soil was observed. It has been concluded that the curing time increased the strength values measured in the UU test, ST test, and UU test. For the UC and ST tests, the most notable improvement in mechanical properties occurred within the first five days of curing, with only slight changes in the period between five and 30 days. For the UU test, changes recorded in the mechanical properties between five and 30 days were considerable. Since the specimens for the UU test were larger than the specimens for the UC and the ST test, it can be concluded that the size of the specimens has an important role in achieving the maximum strength. In other words, larger specimens need more time to dry and to achieve maximum strength.

It was found that the increase in biopolymer concentration enhances the strength of the soil. However, the direct shear test showed that XG, GG, and BG tend to increase the cohesion of the silty sand, but that the friction angle of the treated soil decreases with time. Furthermore, it should be noted that the relation between mechanical properties and biopolymer concentration is not necessarily linear. In other words, each biopolymer-improved soil has an optimum biopolymer concentration. Thus, a high concentration of biopolymers does not guarantee high soil strength. The optimum biopolymer concentration may vary with the biopolymer type, soil type, and water content.

Furthermore, biopolymers proved to be an effective solution to increase the strength of soil in laboratory conditions, but it is still unclear how long they can endure in the natural environment. Comparing the wetting-drying durability to the atmospheric conditions, XG-treated specimens showed higher durability than the specimens made of plain soil, which shows more potential in biopolymers (foremost XG) which should be further researched. The SEM analysis showed the difference in the biopolymer-soil interaction depending on the soil type. It can be concluded that biopolymer links are more exposed in pure sand and that interaction of biopolymers with water will happen faster in that type of soil than in soil with a high concentration of fine particles. This study only scraped the surface regarding the durability of biopolymers in the environment. Therefore, further research should be conducted to investigate in greater detail the durability of biopolymers in relation to environmental influence.

In summary, out of the five different biopolymers which were used for this research, XG, GG, and BG showed the most dominant effect on the improvement of soil strength. For the soil that was used in this research, the optimum biopolymer concentration of XG was close to 2%, whereas the optimum GG concentration was close to 1%. Investigation at higher concentrations of BG is needed to provide an estimate for the optimum BG concentration. However, XG, GG, and BG showed promising results for soil stabilization and provided enormous potential for future sustainable engineering^49,50^.
